# Amplification of the *EGFR* and *CCND1* Are Coordinated and Play Important Roles in the Progression of Oral Squamous Cell Carcinomas

**DOI:** 10.3390/cancers11060760

**Published:** 2019-05-31

**Authors:** Huei-Tzu Chien, Sou-De Cheng, Chun-Ta Liao, Hung-Ming Wang, Shiang-Fu Huang

**Affiliations:** 1 Department of Public Health, Chang Gung University, Tao-Yuan 33302, Taiwan; kathy.htchien@gmail.com; 2 Department of Nutrition and Health Sciences, Chang Gung University of Science and Technology, Tao-Yuan 33302, Taiwan; 3 Department of Anatomy, Chang Gung University, Tao-Yuan 33302, Taiwan; soude@mail.cgu.edu.tw; 4 Department of Otolaryngology, Head and Neck Surgery, Chang Gung Memorial Hospital, Linkou branch, Tao-Yuan 33302, Taiwan; liaoct@adm.cgmh.org.tw; 5 Division of Hematology/Oncology, Department of Internal Medicine, Chang Gung Memorial Hospital, Linkou branch, Tao-Yuan 33302, Taiwan; whm526@adm.cgmh.org.tw

**Keywords:** copy number analysis, amplification, *EGFR*, *CCND1*, oral squamous cell carcinoma

## Abstract

Oral squamous cell carcinoma (OSCC) is a common cancer in Taiwan and worldwide. To provide some clues for clinical management of OSCC, 72 advanced-stage OSCCs were analyzed using two microarray platforms (26 cases with Affymetrix 500 K and 46 cases with Affymetrix SNP 6.0). Genomic identification of significant targets in cancer analyses were used to identify significant copy number alterations (CNAs) using a *q*-value cutoff of 0.25. Among the several significant regions, 12 CNAs were common between these two platforms. Two gain regions contained the well-known oncogenes *EGFR* (7p11.2) and *CCND1* (11q13.3) and several known cancer suppressor genes, such as *FHIT* (3p14.2–p12.1), *FAT1* (4q35.1), *CDKN2A* (9p21.3), and *ATM* (11q22.3–q24.3), reside within the 10 deletion regions. Copy number gains of *EGFR* and *CCND1* were further confirmed by fluorescence in situ hybridization and TaqMan CN assay, respectively, in 257 OSCC cases. Our results indicate that *EGFR* and *CCND1* CNAs are significantly associated with clinical stage, tumor differentiation, and lymph node metastasis. Furthermore, *EGFR* and *CCND1* CNAs have an additive effect on OSCC tumor progression. Thus, current genome-wide CNA analysis provides clues for future characterization of important oncogenes and tumor suppressor genes associated with the behaviors of the disease.

## 1. Introduction

Cancer is complicated in its behavior which is usually related with genomic instability [1]. The genetic alterations are not the same in every tumor cell. Tumor cells that harbor chromosomal aberrations involving important tumor suppressor genes or oncogenes could behave more aggressively and can be used as predictive and prognostic markers [2]. Oral cancer ranked fourth in the incidence of all cancers in Taiwan and is the sixth most common malignancy worldwide [3]. Cigarette, areca quid (AQ), and alcohol are the three main environmental carcinogens for oral squamous cell carcinoma (OSCC) in Taiwan [4,5]. Several studies demonstrated that both tobacco and areca nut extract are toxic to cells and induce genome instability [6,7]. In recent years, new radiation therapy techniques, target therapies and immuno-oncologic agents have been developed in cancer treatment but the advancements in the control of oral cancer were limited. The 5-year survival rate of OSCC has remained almost unchanged at about 50% over the past 30 years [8]. Therefore, we utilized high-throughput genome-wide analysis to improve our understanding of the existing therapies and explored the possibilities for new therapeutics. Copy number alterations (CNAs) are a sign of genome instability and are frequently observed in malignant tumors. CNAs have been extensively investigated in many cancers, and some known biomarkers were identified though these studies (e.g., *EGFR* amplification in non-small cell lung cancer [9]; *erb-b2* amplification in breast cancer [10]). Understanding the genetic alterations and related molecular mechanisms that drive the tumorigenesis and metastasis of OSCCs can help investigators develop new therapeutic strategies and improve the control of oral cancer. The single nucleotide polymorphism (SNP) array has opened up new possibilities to catalogue CNAs at high resolution and throughput [11,12,13]. Several chromosomal aberrations have been identified in previous OSCCs studies, including both loss and gain of chromosomes [14]. Only a minority of these loci involve the true “driver” genes contributing to tumorigenesis and/or tumor progression. The others, considered “passenger” genes, may be altered simply because of their chromosomal location and proximity to the target genes [15]. Thus, identifying true disease-related aberrations may provide clues for the treatment and/or prognosis of OSCC. In the present study, we analyzed 26 and 46 OSCCs on the platforms of Affymetrix 500-K and Affymetrix Genome-Wide Human SNP Array 6.0, respectively. Furthermore, to distinguish important CNAs from random events, we used genomic identification of significant targets in cancer (GISTIC), which considers both the frequency and degree of CNAs [16]. Finally, fluorescence in situ hybridization (FISH) and TaqMan copy number (CN) assays were used for validation. The roles and clinical significance of amplification of the *EGFR* and *CCND1* in OSCC were investigated.

## 2. Results

### 2.1. Identification of Common, Distinct CNAs in 72 OSCCs

High-resolution genomic analyses using 500 K SNP arrays and SNP 6.0 arrays were performed in 26 and 46 cases, respectively (Table 1). GISTIC analyses identified 41 (2 gains and 39 losses) and 32 (4 gains and 28 losses) distinct CNAs from the 500 K and SNP 6.0 platforms, respectively (Tables S1 and S2). The median number of distinct CNAs (gains or losses) per OSCC was 10 (range: 0–27) for the 500 K platform and 7 (range: 0–27) for the SNP 6.0 platform (Figure S1). It is worth noting that similar patterns of CN gains and diverse patterns of CN losses were observed from these two platforms (Figure S2). Based on the position of distinct CNAs estimated from GISTIC, a total of 12 common distinct CNAs (namely gains in 7p11.2 and 11q13.3; losses in 2q23.3–q24.2, 3p14.2–p12.1, 4q35.2, 7q33–q34, 9p21.3, 11q22.3–q24.3, 16q23.1, 18q11.2–q22.3, 21q21.1 and 21q22.3) were identified by these two platforms (Table 2). Among them, the highest frequency (51%, 37/72) of CNAs occurred in 7p11.2 and 11q13.3. To validate CNAs found in the present series of OSCCs, SNP 6.0 array data from another 68 Taiwanese OSCCs were randomly extracted from the Gene Expression Omnibus data repository (accession number GSE25103) [17], processed with GenePattern pipeline; 51 CNAs (8 gains and 43 losses) were observed (Table S3). As indicated in Table 2, 9 (75%, two gains and seven losses) of the 12 common CNAs were also identified in this dataset; this confirms that CN gains in 7p11.2 and 11q13.3 were common in Taiwanese OSCCs.

### 2.2. Confirmation of EGFR and CCND1, Including in the CNA Region of 7p11.2 and 11q13.3

As stated above, there were two common CN gains (7p11.2 and 11q13.3) refined in GISTIC analyses. Based on the GISTIC high-peak region, the CN gain in 7p11.2 (spanning 54.62 to 55.86 Mb) contained *EGFR* and *SEC61G*, and the CN gain in 11q13.3 (spanning 68.96 to 70.32 Mb) contained *MYEOV*, *CCND1*, *ORAOV1*, *FADD*, and *CTTN*. *EGFR* is the most likely candidate driver gene in 7p11.2 involved in OSCC molecular pathogenesis [18]. The cell cycle regulatory gene *CCND1* located in 11q13.3 is a downstream effector of *EGFR* and is commonly deregulated in various cancers including head and neck cancer. Moreover, several studies have reported that *CCND1* amplification and overexpression are associated with poor prognosis, cisplatin resistance, and EGFR-inhibitor resistance [19,20]. Thus, these two genes were further validated using FISH (*EGFR*) or TaqMan CN assays (*CCND1*). The validation rate was 75% for *EGFR* and 67% for *CCND1* (Figure S3).

### 2.3. Clinical Implications of CNA’s in EGFR and CCND1

To further explore the clinical value of common CN gains of *EGFR* and *CCND1* in OSCC, 257 OSCC cases were included for further analysis (Table 1). Of these, 27 (11%) and 52 (20%) OSCCs displayed *EGFR* polysomy and amplification, respectively, and 135 (53%) displayed *CCND1* CN amplification (Table 3). In addition, as shown in Table 3, *EGFR* CNA’s (polysomy or amplification) were associated with young age (chi-square trend test, *p* = 0.04), advanced tumor stage (*p* = 0.01), a higher grade of tumor differentiation (*p* = 0.03), and LNM (*p* < 0.01), but not with tumor site, AQ chewing, cigarette smoking, or alcohol drinking. *CCND1* CN amplification was associated with advanced tumor stage (*p* = 0.04), a higher grade of tumor differentiation (*p* < 0.01), LNM (*p* < 0.01), and alcohol drinking (*p* = 0.03), but not age, the tumor site, AQ chewing, or cigarette smoking. On the other hand, the CNA frequency of *EGFR* was 31% (79/257), and the proportion of *CCND1* amplification was 53% (135/257). Both the *EGFR* and *CCND1* were altered in 22% (56/257) of OSCC samples (Table S4). When we considered the statuses of both genes, the synergistic effects of *EGFR* and *CCND1* were associated with tumor stage (*p* < 0.01), LNM (*p* < 0.01), and tumor differentiation (*p* < 0.01) (Table 3).

### 2.4. Prognostic Implications of CNA’s with EGFR and CCND1

**Figure 1:**
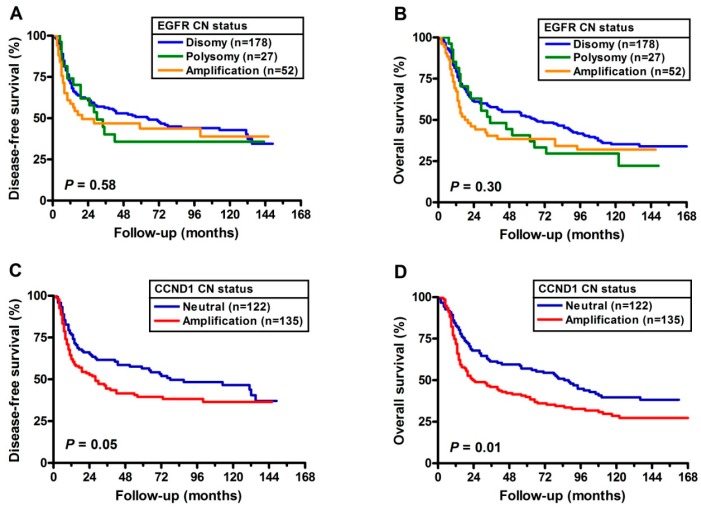
Kaplan–Meier curves for disease-free survival (DFS) and overall survival (OS) based on analysis of *EGFR* gene copies (**A**,**B**) and *CCND1* gene copies (**C**,**D**). Patients with *CCND1* gene amplification had significantly worse DFS (*p* = 0.05) and OS (*p* = 0.01).

**Figure 2:**
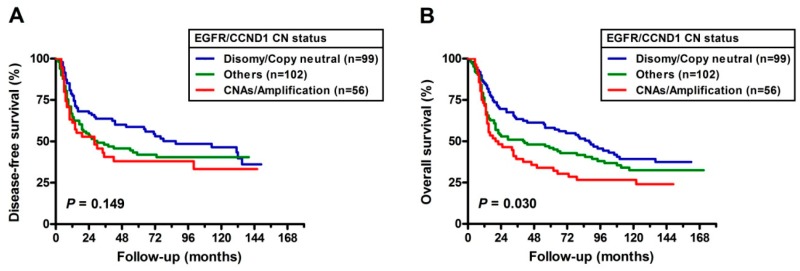
Additive effects of *EGFR* and *CCND1* copy number changes observed in DFS (**A**) and OS (**B**).

Kaplan-Meier survival curves for DFS and overall survival (OS) are presented in Figure 1. Although not statistically significant, the three *EGFR* FISH patterns with median time to relapse were 64.00, 30.00, and 20.00 months. In addition, the median times to death were 89.00, 46.00, and 33.00 months. On the other hand, as shown in panels C and D, the median DFS in those with *CCND1* CN neutral were 78.00 months and those with *CCND1* CN amplification were 28 months. The median OS in those with *CCND1* CN neutral were 102.00 months and those with *CCND1* CN amplification were 41.00 months. The curves demonstrate the adverse impact of *CCND1* CN amplification on both DFS (borderline, *p* = 0.05) and OS (*p* = 0.01). Furthermore, the OSCC patients with the worst prognoses had both the *EGFR* and *CCND1* with CNAs (hazards ratio (HR) = 1.750, 95% CI = 1.126–2.719) (Table 4). The median DFS in those having both *EGFR* and *CCND1* CN neutral, either *EGFR* or *CCND1* amplification and both *EGFR*/*CCND1* CN amplification were 87.00 months, 30.00 months, and 28.00 months respectively. The median OS in those having both *EGFR* and *CCND1* CN neutral, either *EGFR* or *CCND1* amplification and both *EGFR*/*CCND1* CN amplification were 102.00 months, 65.00 months, and 33.00 months respectively (Figure 2).

## 3. Discussion

Initially, the experiments were performed on 500-K SNP arrays followed by SNP 6.0 arrays, which have a much higher resolution for the detection of CNAs (with more than 1.8 million markers). GISTIC analyses can determine CNAs associated with OSCC from potentially random events and organizes the profiles of both platforms. The two platforms reveal several CNAs and individually highlight genomic regions that are most likely to encode oncogenes and tumor suppressor genes (Tables S1 and S2). To identify the clinical significance of CNAs, regions of common CNAs were identified in 72 OSCC tumors on two different platforms (Table 2). There were several established cancer genes involved in a total of 12 common CNAs identified according to GISTIC, including *EGFR* [21], *CCND1* [22], *FHIT* [23], and *FAT1* [24]. These data demonstrate that the GISTIC method was reliable despite the small sample size. The amplicons at 7p11.2 and 11q13.3 were the top two CNAs. These findings are similar to observations of CNAs from 29 OSCCs in a study in Taiwan that used the 250K SNP array method [18]. The higher CNA frequency of 7p11.2 (51% vs. 31%) and 11q13.3 (51% vs. 17%) in our study might be due to the increasing density of the SNP array (500-K SNP array and SNP 6.0). *EGFR* and *CCND1* were the GISTIC-identified CNA target genes in the top two CNA peaks. *EGFR* is the candidate gene of the 7p11.2 region and regulates many cellular functions including cell proliferation and survival through tyrosine kinase downstream signaling such as the PI3K-AKT pathway or STAT3 activation [25]. *CCND1* is a proto-oncogene that encodes cyclin D1, which is a key regulator of the G1 phase of the cell cycle [26]. Cyclin D1 binds and activates *CDK4* and *CDK6*, and this complex catalyzes Rb protein phosphorylation resulting in the release of transcriptional regulators E2F from Rb, which promotes cell cycle progression [26]. The role of *CCND1* had been investigated in both HNSCCs and oral cancers. The results indicated *CCND1* plays an important role in the tumor progression in OSCC and is a prognostic marker [20,27]. Interestingly, these two major genes are also significant in a published dataset in Taiwan [17]. These findings highlight the critical role of *EGFR* and *CCND1* in OSCC in Taiwan.

Furthermore, we used two different approaches to verify the CN of *EGFR* and *CCND1*. Using the FISH method, the frequency of *EGFR* changes (polysomy or amplification) was 31% (79/257). The frequency of *EGFR* CNAs has been reported to be 18–44% [14,28,29]. The large range might be due to differences in tumor location and tumor stage. *EGFR* amplification is also frequently found in many cancers such as lung cancer, breast cancer, and glioblastomas [30]. CN amplification is associated with LNM and poorer prognoses in HNSCC [28,31]. In our study, the increased *EGFR* gene copies showed a higher risk of LNM (OR = 2.368, 95% CI = 1.372–4.026, *p* < 0.01). Comparing the median survival times, there was a definite trend toward worse survival in cases with increased *EGFR* gene copies, although the association between *EGFR* amplification and survival was nonsignificant. According to a previous study, *EGFR* gene amplification is highly correlated with overexpression of *EGFR* protein [32]. *EGFR* expression is also correlated with lower histologic tumor differentiation [33]. In this study, we found a similar association between tumor differentiation and *EGFR* CNAs.

The frequency of *CCND1* amplification (135/257, 53%) found in this study was similar to a previous OSCC study in Taiwan (41/82, 50%) [22]. We found that *CCND1* gene amplification significantly correlated with poorer differentiation and LNM. Liu et al. and Myo et al. have shown that *CCND1* CNA are related with cyclin D1 protein overexpression [22,34]. In a previous study, we noted the overexpression of *CCND1* protein in 37% of OSCC cases and found that this was correlated with tumor differentiation, LNM, and poor clinical outcomes. Thus, *CCND1* amplification may indirectly contribute to the acquisition of invasive ability and metastatic potential. Another explanation of this observation is that *CCND1* amplification might reflect general genomic instability in cancer cells, and such cells possess a more aggressive phenotype [35]. Smoking, alcohol drinking, and AQ chewing are well-known risk factors for OSCC. Alcoholic drinks might act as a solvent for the penetration of carcinogens through the mucosa of the upper aerodigestive organ [36]. Furthermore, research on alcohol and cancer remains limited in terms of clinical, epidemiological, and experimental settings. Although only borderline, alcohol consumption increased the risk for *CCND1* CNA (relative risk (RR) = 1.860, 95% CI = 1.011–3.422) in our study (Table S5). Recently, Urashima et al. found that several somatic CNAs were associated with heavy alcohol consumption including *CCND1* amplification (heavy drinkers vs. moderate/non-drinkers, RR = 1.94, 95% CI = 1.12–3.37, *p* = 0.019) [37]. The presence of ECS is a marker for a biologically-aggressive disease and is the most pivotal predictor of survival, recurrence, and distant metastasis [38]. Michikawa et al., indicated that the identification of numerical aberrations in *EGFR* might be a more useful tool for selecting patients at high risk for ECS compared to *CCND1* aberrations [38]. However, we previously found a positive association between the overexpression of CCND1 protein and ECS [39]. We might need a large cohort to further clarify the correlation between *CCND1* amplification and ECS status (Table 3). In this study, *CCND1* CNAs were significantly associated with reduced DFS and OS, and these observations were highly correlated with LNM.

*CCND1* plays a crucial role in canonical or non-canonical pathways of *EGFR* [40]. Furthermore, several in vitro studies have shown that deregulated *CCND1* overexpression is significantly associated with resistance of HNSCC to EGFR inhibitors. These observations suggest that *CCND1* is a pivotal downstream target gene in *EGFR*-driven tumorigenesis. Therefore, the genetic statuses of not only *EGFR* but also *CCND1* are needed to predict the therapeutic effects of *EGFR* inhibitors [19,41]. In our observations, OSCC patients with two concomitant events have the worst prognosis (Figure 2B, Table 4). Moreover, the rate of co-alterations in both genes was 22% (56/257), higher than in a previous study (10%, 3/29) [18]. However, this finding still suggests that there are other downstream targets for *EGFR* and other upstream regulators for *CCND1*. These findings may have important therapeutic implications for OSCC patients.

## 4. Materials and Methods

### 4.1. Patients, Specimens and Clinical Diagnosis

This study was approved by the Institutional Review Board of Chang Gung Medical Foundation, Lin-Kou, Taiwan (IRB#102-5616B). All patients included in the study received primary radical surgery at this hospital between 1999 and 2004. The methods in this study were carried out in accordance with the relevant guidelines, including any relevant details. All patients gave written informed consent before participating, and information regarding current and past cigarette smoking, alcohol drinking, and AQ chewing habits was obtained. All cases were histologically confirmed and scored according to the recommendations for the reporting of specimens containing oral cavity, oropharynx, or hypopharynx neoplasms by the Association of Directors of Anatomical and Surgical Pathology (ADASP) (2000). For each case, tumor tissues were taken, dissected into small pieces, frozen immediately in liquid nitrogen, and stored at −80 °C. Ten milliliters of venous blood was separated into plasma, buffy coat cells, and red blood cells by centrifugation within 18 h of obtaining the blood. Then the buffy coat cells were stored at −80 °C. Genomic DNA from the tissue and buffy coat were purified as previously described [42]. Because the sex ratio (male versus female) of OSCC incidence in Taiwan was ~10.4 after adjusting for age, only male patients were included. High-resolution genomic analyses using 500-K SNP arrays and SNP 6.0 arrays were performed in 26 and 46 cases, respectively. In addition, a total of 257 OSCCs were included for further EGFR and *CCND1* CN analysis (Table 1).

### 4.2. High-Resolution SNP Array and Data Analysis

A total of 500 ng genomic DNA from each OSCC were subjected to SNP genotyping with either the Human Mapping 500-K Array set or Genome-Wide Human SNP Array 6.0 (Affymetrix Inc, Santa Clara, CA, USA). Genomic DNA preparation and chip processing were performed according to Affymetrix’s recommended protocols. Genotyping was performed at the National Center for Genome Medicine (NCGM) t Academia Sinica, Taipei, Taiwan. Array image data were preliminarily analyzed with Genotyping Console 4.0 to derive the CEL files. The CEL files of the 500-K Array were processed using Partek Genomic Suite (Partek Inc., Chesterfield, MO, USA), and CNs were created from allele intensities, using 270 CN samples from the HapMap collection as the baseline. Log ratio intensities were adjusted for local GC content in unpaired samples to decrease genomic waviness, and CNA segments (gains and losses) were built using a Hidden Markov Model with a minimum of four genomic markers per CNA segment. The CEL files of SNP Array 6.0 and 45 HapMap Han Chinese data were processed using a GenePattern pipeline that runs the following modules: SNPFileCreator_SNP6, CopyNumberInference, RemoveCopyNumberOutliers, TangentNormalization, and ParallelCBS. Distinct, recurrent CNA from the above two platforms were identified by GISTIC 2.0 using a Web-based interface (http://genepattern.broadinstitute.org/) with CNA thresholds of +0.1, a join segment size of four markers, and a *q*-value threshold of 0.25 [43]. The naturally occurring CNVs presented in the Database of Genomic Variants (DGV) were excluded during GISTIC 2.0 processing. SNP, gene, and cytogenetic band locations were based on GRCh37/hg19. To validate the CNAs found in our study, SNP 6.0 array data of 68 Taiwanese OSCCs were randomly extracted from the Gene Expression Omnibus data repository (accession number GSE25103) [17] and further processed using GenePattern pipeline as described above.

### 4.3. Fluorescence In Situ Hybridization (FISH) for EGFR

FISH analyses were performed as previously described using the LSI *EGFR* SpectrumOrange/CEP 7 SpectrumGreen probe system (Vysis; Abbott Laboratories, Downers Grove, IL, USA) [32]. At least 100 non-overlapping nuclei per case were scored independently by two independent observers. *EGFR* FISH patterns were classified as follows: normal disomy, with ≤2 copies in more than 90% of analyzed cells; polysomy, ≥3 copies in more than 40% of analyzed cells; and amplification defined by the presence of tight *EGFR* clusters in ≥10% of analyzed cells [32].

### 4.4. TaqMan CN Assays via Quantitative Real-Time Polymerase Chain Reaction (qPCR) for CCND1

Next, qPCR analyses were performed according to the Minimum Information for Publication of Quantitative Real-Time PCR Experiments (MIQE) guidelines [44]. CCND1 CN was measured using a duplex TaqMan Gene Copy Number Assay (Applied Biosystems). Two hydrolysis probes were selected, in exon 1 (Hs01818912_cn) and exon 5 (Hs02353610_cn), using *RNase P* as a reference for the duplex assay. CN assays were performed in triplicate using TaqMan Genotyping Master Mix in the 7500 Fast Real-Time PCR System (Applied Biosystems, Foster City, CA, USA). Thermal cycling conditions included initial denaturation at 95 °C for 10 min, followed by 40 cycles of 15 s at 95 °C for denaturing and 60 s at 60 °C for annealing and extension. Two known control samples from peripheral blood (carrying two alleles) were included in each reaction plate. The comparative quantification cycle (∆∆Cq) method was used for data analysis and >0.59 was set as the cutoff value for amplification [21].

## 5. Conclusions

In the present study, we constructed a CNA profile of OSCC in Taiwan using a high-density SNP array and GISTIC. Furthermore, we validated the alterations in *EGFR* and *CCND1* and demonstrated the clinical implications of these two candidate genes.

## Figures and Tables

**Table 1:** Clinicopathological characteristics of the OSCCs patients studied.

Characteristics	500 K Array (*n* = 26)	SNP 6.0 Array (*n* = 46)	257 OSCC Cases for Further Analysis
Age (years)			
Mean ± SD	48.15 ± 11.39	50.41 ± 10.79	49.95 ± 11.23
Range	27–71	30–74	26–78
Site of primary tumor [*N* (%)]			
Tongue	11 (42)	26 (57)	90 (35)
Bucca	15 (58)	20 (43)	94 (37)
Others	-	-	73 (28)
Clinical stage [*N* (%)]			
I/II	2 (8)	0 (0)	65 (25)
III/IV	24 (92)	46 (100)	192 (75)
Primary tumor status [*N* (%)]			
T1/T2	9 (35)	18 (39)	121 (47)
T3/T4	17 (65)	28 (61)	136 (53)
Lymph node metastasis [*N* (%)]			
No	8 (31)	10 (22)	132 (51)
Yes	18 (69)	36 (78)	125 (49)
Extra-capsular spread [*N* (%)] ^a^			
Yes	14 (78)	26 (72)	76 (61)
No	4 (22)	10 (28)	48 (39)
Tumor differentiation [*N* (%)]			
Well differentiated	9 (35)	25 (35)	98 (38)
Moderately/Poorly differentiated	17 (65)	47 (65)	159 (62)
AQ chewing [*N* (%)]			
Yes	15 (58)	36 (78)	223 (87)
No	11 (42)	10 (22)	34 (13)
Cigarette smoking [*N* (%)]			
Yes	11 (42)	35 (76)	220 (86)
No	15 (58)	11 (24)	37 (14)
Alcohol drinking [*N* (%)]			
Yes	14 (54)	20 (43)	138 (54)
No	12 (46)	26 (57)	119 (46)

AQ: areca quid; ^a^ One patient lacked the extra-capsular spread data.

**Table 2:** Summary of the 12 common CNAs obtained from 500 K and SNP 6.0 platform.

Cytogenetic Loci	GISTIC Wide Peak Region (Mb ^a^)	Size (Mb)	Total Frequency (%, *n* = 72)	Cases with CNAs by 500-K Platform (*n* = 26)	Cases with CNAs by SNP 6.0 Platform (*n* = 46)	Candidate Genes
Gains						
7p11.2 ^b^	54.62–55.86	1.24	51	14	23	*SEC61G*, *EGFR*
11q13.3 ^b^	68.96–70.32	1.36	51	14	23	*MYEOV1*, *CCND1*, *ORAOV1*, *FADD*, *CTTN*
Losses						
2q23.3–q24.2 ^b^	124.78–243.20	118.24	10	3	4	*ING5*, *LRP1B*, *DAPL1*
3p14.2–p12.1	25.64–93.78	68.14	42	12	18	*FHIT*, *MLH1*, *BAP1*, *SETD2*, *PBRM1*
4q35.2 ^b^	187.48–188.24	0.76	33	8	16	*FAT1*
7q33–q34 ^b^	97.61–159.14	63.91	18	6	7	*MIR335*, *ING3*
9p21.3 ^b^	21.56–22.00	0.44	26	4	15	*CDKN2A*
11q22.3–q24.3 ^b^	72.39–135.01	62.62	38	10	17	*ATM*, *MRE11A*, *CHEK1*, *H2AFX*
16q23.1	75.46–79.63	4.17	11	2	6	*WWOX*
18q11.2–q22.3 ^b^	18.69–78.08	59.39	40	9	20	*DCC*
21q21.1	10.19–29.10	18.91	33	7	17	*CHODL*
21q22.3 ^b^	44.30–48.13	3.83	33	6	18	*NDUFV3*

^a^ Mb: mega base; ^b^ CNAs also identified when validating the dataset extracted from the Gene Expression Omnibus data repository (accession number GSE25103).

**Table 3:** Associations between *EGFR*/*CCND1* copy number and clinicopathological parameters.

	*EGFR*				*CCND1*			*EGFR/CCND1*			
	Di	Poly	Amp	*P* Value	*N*	Amp	*P* Value	Di/Neu	Others	Amp/CNA	*P* Value
Age (years)											
<50	90 (64)	17 (12)	34 (24)	0.11	63 (45)	78 (55)	0.32	51 (36)	51 (36)	39 (28)	0.04
≥50	88 (76)	10 (9)	18 (16)	0.04^a^	59 (51)	57 (49)		48 (41)	51 (44)	17 (15)	0.06 ^a^
Subsites											
Tongue	63 (70)	9 (10)	18 (20)	0.97	41 (46)	49 (54)	0.90	38 (42)	28 (31)	24 (27)	0.24
Bucca	63 (67)	10 (11)	21 (22)	0.82 ^a^	46 (49)	48 (51)		35 (37)	39 (41)	20 (21)	0.77 ^a^
Others	52 (71)	8 (11)	13 (18)		35 (48)	38 (52)		26 (36)	35 (48)	12 (16)	
Clinical stage											
I/II	52 (80)	8 (12)	5 (8)	0.01	38 (58)	27 (44)	0.04	33 (51)	24 (37)	8 (12)	0.03
III/IV	126 (66)	19 (10)	47 (24)	<0.01 ^a^	84 (44)	108 (56)		66 (34)	78 (41)	48 (25)	<0.01^a^
Primary tumor status											
T1/T2	90 (74)	12 (10)	19 (16)	0.20	60 (50)	61 (50)	0.52	51 (42)	48 (40)	22 (18)	0.34
T3/T4	88 (65)	15 (11)	33 (24)	0.07 ^a^	62 (46)	74 (54)		48 (35)	54 (40)	34 (25)	0.15 ^a^
LNM ^c^											
No	103 (78)	14 (11)	15 (11)	<0.01	80 (61)	52 (39)	<0.01	67 (51)	49 (37)	16 (12)	<0.01
Yes	75 (60)	13 (10)	37 (30)	<0.01 ^a^	42 (34)	83 (66)		32 (26)	53 (42)	40 (32)	<0.01^a^
ECS^d^											
Yes	45 (59)	9 (12)	22 (29)	0.82	32 (42)	44 (58)	0.02	23 (30)	31 (41)	22 (29)	0.33
No	29 (60)	4 (8)	15 (31)	0.95 ^a^	10 (21)	38 (79)		9 (19)	21 (44)	18 (38)	0.15 ^a^
Tumor differentiation											
Well	77 (79)	9 (9)	12 (12)	0.03	63 (64)	36 (36)	<0.01	57 (58)	27 (27)	15 (15)	<0.01
Mod/Poor	101 (64)	18 (11)	40 (25)	<0.01 ^a^	59 (38)	98 (62)		42 (27)	74 (47)	41 (26)	<0.01 ^a^
AQ ^e^ chewing											
Yes	156 (70)	21 (9)	46 (21)	0.34	110 (49)	113 (51)	0.13	89 (40)	88 (39)	46 (21)	0.39
No	22 (65)	6 (18)	6 (18)	0.88 ^a^	12 (35)	22 (65)		10 (29)	14 (41)	10 (29)	0.17 ^a^
Cigarette smoking											
Yes	153 (70)	23 (10)	44 (20)	0.97	107 (49)	113 (51)	0.36	86 (39)	88 (40)	46 (21)	0.70
No	25 (68)	4 (11)	8 (22)	0.80 ^a^	15 (41)	22 (59)		13 (35)	14 (38)	10 (27)	0.46 ^a^
Alcohol drinking											
Yes	96 (70)	14 (10)	28 (20)	0.98	57 (41)	81 (59)	0.03	46 (33)	61 (44)	31 (22)	0.16
No	82 (69)	13 (11)	24 (20)	0.96^a^	65 (55)	54 (45)		53 (45)	41 (34)	25 (21)	0.18^a^

^a^ By chi-square trend test; ^b^ *EGFR* CNA’s contain *EGFR* polysomy or *EGFR* amplification cases; ^c^ LNM: lymph node metastasis; ^d^ ECS: extra-capsular spread; ^e^ AQ: areca quid; Di: disomy; Poly: polysomy; Amp: amplification; Neu: copy number neutral.

**Table 4:** Univariate Cox proportional hazard analysis of prognostic covariates in 257 patients with OSCC regarding disease-free survival and overall survival.

	Disease-Free Survival		Overall Survival	
Characteristic	HR (95% CI)	*P* Value	HR (95% CI)	*P* Value
Age (yrs)				
<50	1		1	
> = 50	0.901 (0.641–1.265)	0.55	1.198 (0.883–1.626)	0.25
Tumor differentiation				
Well differentiated	1		1	
Moderately/poorly differentiated	1.282 (0.903–1.821)	0.17	1.258 (0.915–1.729)	0.16
Primary tumor status				
T1/T2	1		1	
T3/T4	1.104 (0.788–1.546)	0.57	1.487 (1.092–2.025)	**0.01**
Lymph node status				
LNM ^a^−, ECS ^b^−	1		1	
LNM+, ECS−	1.256 (0.777–2.030)	0.35	1.643 (1.083–2.493)	**0.02**
LNM+, ECS+	2.354 (1.621–3.420)	**<0.01**	2.360 (1.673–3.329)	**<0.01**
*EGFR* copy number				
Disomy	1		1	
Polysomy	1.160 (0.682–1.973)	0.58	1.358 (0.819–2.253)	0.24
Amplification	1.231 (0.802–1.891)	0.34	1.264 (0.827–1.931)	0.28
*CCND1* copy number				
Neutral	1		1	
Amplification	1.402 (0.998–1.971)	0.05	1.485 (1.091–2.021)	**0.01**
*EGFR* and *CCND1* CN status				
*EGFR* disomy/*CCND1* copy neutral	1		1	
Others	1.332 (0.905–1.961)	0.15	1.368 (0.926–2.022)	0.12
*EGFR* CNAs ^c^ and *CCND1* amplification	1.501 (0.960–2.347)	0.08	1.750 (1.126–2.719)	**0.01**

^a^ LNM: lymph node metastasis; ^b^ ECS: extra-capsular spread; ^c^ Subgroup of *EGFR* CNAs contains *EGFR* polysomy or amplification OSCC cases.

## Supplementary Materials

The following are available online at https://www.mdpi.com/2072-6694/11/6/760/s1, Figure S1: Distribution of the number of CNAs (events) per sample, Figure S2: Circos plots of CNA’s detected in 72 OSCCs by GISTIC, Figure S3: Validation of *EGFR* and *CCND1* genes copy number alterations, Table S1: Distinct copy number gains and losses identified from 26 OSCCs using 500-K array platform, Table S2: Distinct copy number gains and losses identified from 46 OSCCs using SNP 6.0 platform, Table S3: Distinct copy number gains and losses identified from 68 OSCCs randomly extracted from GSE25103 data repository, Table S4: Association between *EGFR* and *CCND1* genes CN status and Table S5: Association between *CCND1* CN status and cigarette smoking, AQ chewing and alcohol drinking.
