# Supplementary material for: Amplification of the EGFR and CCND1 Are Coordinated and Play Important Roles in the Progression of Oral Squamous Cell Carcinomas
Source: Cancers (Basel). 2019 May 31;11(6):760. doi: 10.3390/cancers11060760 (PMC6627096; doi:10.3390/cancers11060760)
Supplement: Supplementary file 1 [file cancers-11-00760-s001.pdf]

Amplification of the *EGFR* and *CCND1* are Coordinated and Play Important Roles in the Progression of Oral Squamous Cell Carcinomas

Huei-Tzu Chien, Sou-De Cheng, Chun-Ta Liao, Hung-Ming Wang and Shiang-Fu Huang

Supplementary Material

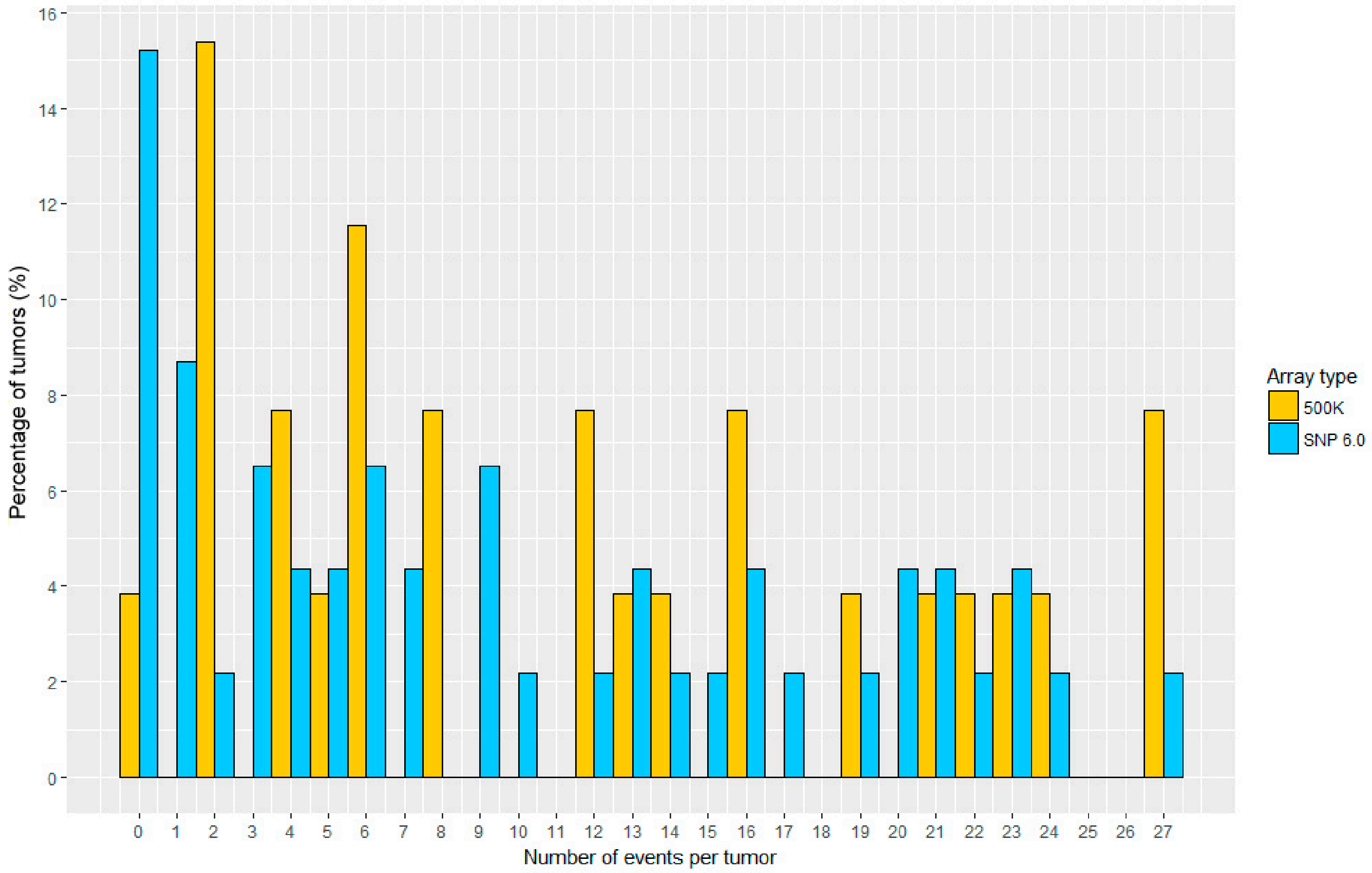

Figure S1. Distribution of the number of CNAs (events) per sample. Results are shown for the 500K array and Array SNP 6.0.

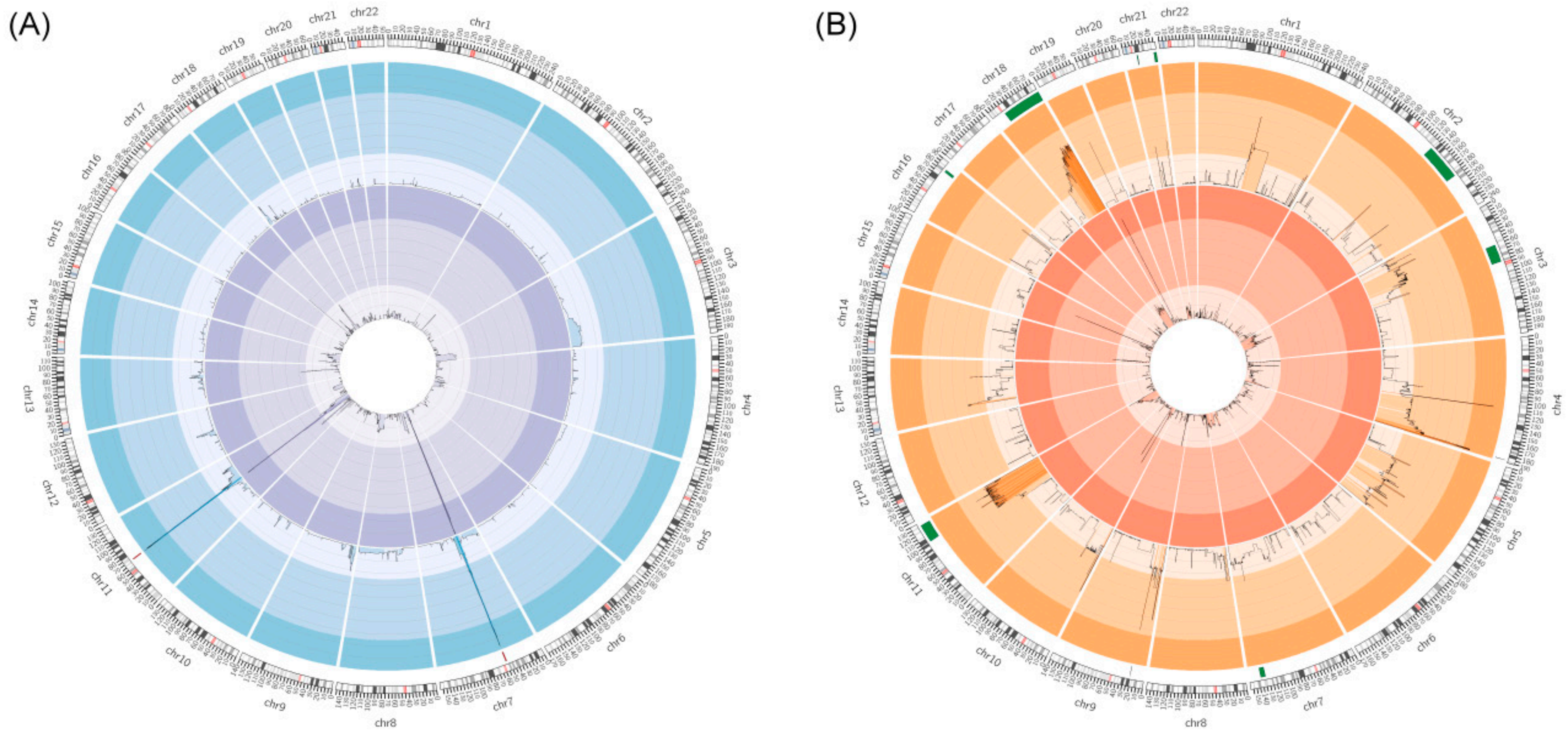

Figure S2. Circos plots of CNAs detected in 72 OSCCs by GISTIC. Chromosome bands and location are shown in outer rings. The curves indicate the G score in amplifications (**A**) and deletions (**B**). The results of SNP 500K are shown in the inner purple and pink rings, the results of SNP 6.0 are shown in the outer blue and orange rings. The red and green bars indicate 12 common CNAs. The two common CNAs in the amplification plot are the *EGFR* (7p11.2) and *CCND1* (11q13.3) gene locations.

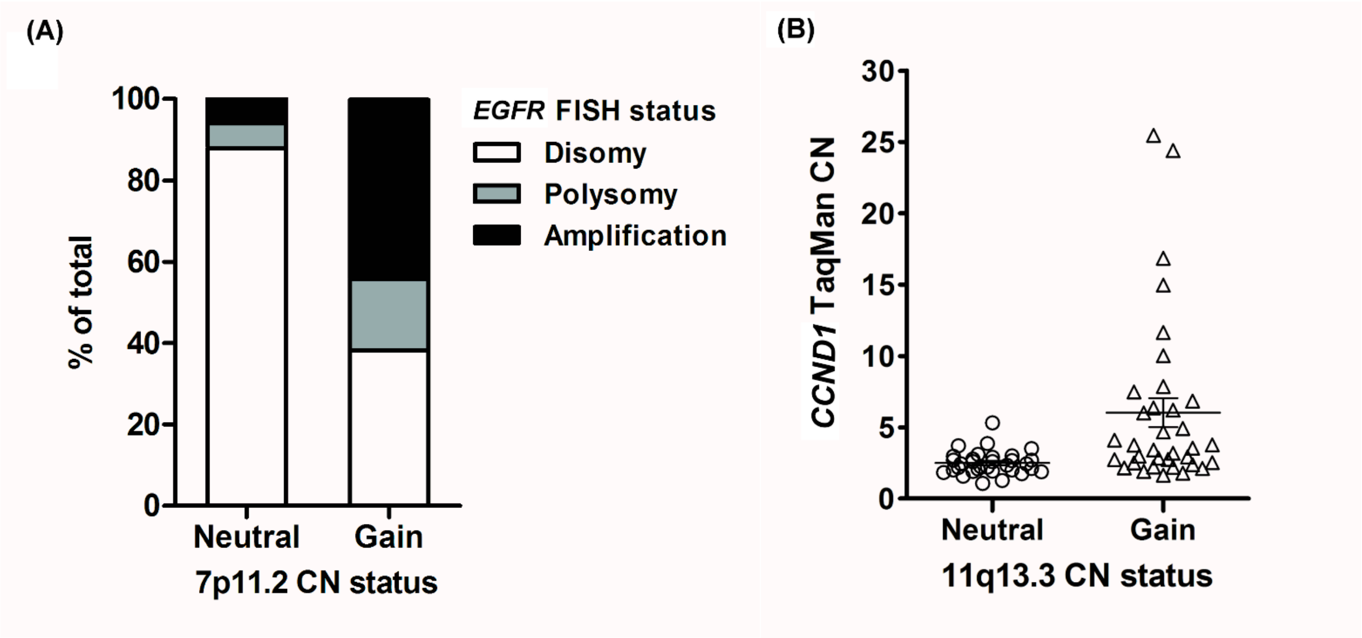

Figure S3. Validation of *EGFR* and *CCND1* genes copy number alterations. The validation of 7p11.2 CN status with *EGFR* gene copy number alterations (**A**), and 11q13.3 CN status with *CCND1* gene copy number (**B**).

Table S1. Distinct copy number gains and losses identified from 26 OSCCs using 500K array platform.

Cytogenetic Loci	GISTIC Wide Peak Region (Mb[a])	Size (Mb[a])	*q*-Value	GISTIC *q*-Value	Frequency (%)	No. of Genes	Candidate Genes
			Gains				
7p11.2	54.62–55.86	1.24	0.016951	0.016951	53.85	7	*SEC61G, EGFR*
11q13.3	68.96–70.07	1.11	0.027864	0.027864	53.85	8	*MYEOV, CCND1, ORAOV1, FADD*
			Losses				
1p32.2	57.17–57.32	0.15	0.12283	0.12283	3.85	1	
1q23.2	160.11–160.18	0.07	0.025834	0.025834	15.38	2	
2q23.3	124.78–243.20	118.24	0.1552	0.19111	11.54	730	*ING5, LRP1B, DAPL1*
2q37.1	234.98–235.89	0.91	0.03283	0.049553	23.08	1	
3p26.1	0–198.02	198.02	0.056468	0.21181	46.15	1276	*FHIT, MLH1, BAP1, SETD2, PBRM1*
3p25.1	15.90–16.22	0.32	0.020471	0.051438	46.15	1	
3p14.2	59.03–61.56	2.53	0.001605	0.0043938	46.15	1	*FHIT*
3p13	70.01–71.81	1.8	0.018395	0.10641	42.31	5	*FOXP1*
4p16.1	9.78–10.08	0.3	0.000324	0.00032384	11.54	2	

4p14	37.95– 38.62	0.67	0.000779	0.0007788	46.15	2	*PTTG2*
4q21.1	77.32–78.43	1.11	0.06449	0.066479	19.23	6	
4q35.2	187.48–188.24	0.76	0.00825	0.0082501	30.77	1	*FAT1*
5q22.2	112.82–113.70	0.88	0.11882	0.10641	19.23	1	
6p22.1	29.95–29.98	0.03	0.025834	0.025834	11.54	1	
6q22.33	128.83–129.90	1.07	0.13407	0.13614	7.69	1	
7q11.22	66.03–75.12	9.09	0.10278	0.12283	15.38	72	
7q34	97.61–159.14	61.53	0.094196	0.10641	23.08	540	*MIR335, ING3*
8p23.3	0–0.6	0.6	0.13407	0.13614	50.00	5	
8p23.2	2.09–6.27	4.18	0.015837	0.015837	57.69	2	*CSMD1*
9p22.2	17.50–18.47	0.97	0.000324	0.00032384	15.38	1	*SH3GL2*
9p21.3	21.56–22.00	0.44	0.17047	0.1552	15.38	3	*CDKN2A*
10p12.1	24.84–25.15	0.31	2.44E-12	2.44E-12	26.92	1	*ARHGAP21*
10q11.21	44.01–44.10	0.09	2.39E-10	2.39E-10	15.38	1	
11q24.3	130.18–130.74	0.56	0.001145	0.0011447	38.46	2	
14q11.2	0–19.55	19.55	5.43E-05	0.00021774	42.31	2	
14q11.2	21.50–21.87	0.37	0.00434	0.015837	34.62	14	
15q14	37.39–38.55	1.16	0.14679	0.14679	15.38	1	
15q15.3	44.15–44.58	0.43	0.020471	0.020471	11.54	2	
15q21.3	57.57–57.67	0.1	2.30E-18	2.30E-18	53.85	1	
16q23.1	75.46–75.51	0.5	0.10129	0.094196	7.69	1	
17q12	36.10–36.59	0.49	0.17999	0.19111	11.54	7	
18q11.2	0–78.08	78.08	0.091647	0.28995	23.08	342	*DCC*
18q11.2	18.69–78.08	59.39	0.022731	0.1552	26.92	253	*DCC*
18q12.1	32.38–32.45	0.07	0.00434	0.031519	30.77	1	
18q21.1	20.83–78.08	57.25	0.00825	0.094196	30.77	238	*DCC*
19p13.11	17.54–17.64	0.1	1.92E-29	1.92E-29	50.00	4	
21q21.1	10.19–29.10	18.91	0.16022	0.1552	26.92	56	*CHODL*
21q22.3	44.59–45.53	0.94	0.065272	0.084587	23.08	11	
21q22.3	46.65–48.13	1.48	0.18257	0.2485	23.08	21	

[a]Mb: mega base pair.

Table S2. Distinct copy number gains and losses identified from 46 OSCCs using SNP 6.0 platform.

Cytogenetic Loci	GISTIC Wide Peak Region (Mb[a])	Size (Mb)	*q*-Value	GISTIC *q*-Value	Frequency (%)	No. of Genes	Candidate Genes
				Gains			
7p11.2	54.30–54.35	0.05	2.41E-12	0.00030515	45.65	0	
7p11.2	55.06–55.27	0.21	1.56E-13	1.09E-08	50.00	1	*EGFR*
11q13.3	69.03–69.17	0.14	1.56E-13	1.96E-08	47.83	1	*MYEOV*
11q13.3	70.11–70.32	0.21	1.56E-13	2.70E-07	47.83	4	*CTTN*
				Losses			
1p13.2	112.30–112.94	0.64	0.029884	0.18362	10.87	2	
1p13.1	116.92– 116.95	0.03	0.00041581	0.003203	15.22	1	
1q43	0–249.25	249.25	0.24251	0.24251	8.70	2410	*CDKN2C, FCGR2B, MUTYH, MYCL1, RPL5, SDHB, ARID1A, TNFRSF14, CAMTA1, SLC45A3*
1q44	245.28–245.91	0.63	0.18362	0.18362	6.52	1	
2q21.2	133.42–187.56	54.14	0.012241	0.01224	8.70	227	*ING5, LRP1B, DAPL1*
3p25.2	12.39–12.53	0.14	0.0054874	0.080113	50.00	1	*PPARG*
3p24.2	24.56–25.65	0.09	0.0045225	0.028436	50.00	2	*RARB*
3p12.1	25.64–93.78	68.14	0.13612	0.12649	39.13	453	*FHIT, MLH1, BAP1, SETD2, PBRM1*
4q25	109.08–109.54	0.46	1.5071E-06	1.62E-06	23.91	2	
4q35.2	187.48–188.23	0.75	1.7569E-06	1.3E-05	36.96	1	*FAT1*
5q11.2	44.81–110.41	65.6	0.022564	0.080113	26.09	265	
5q23.1	118.97–121.19	2.22	0.0032652	0.040879	30.43	2	
5q33.3	61.87–180.92	119.05	0.016923	0.12649	26.09	806	
7q33	95.23–159.14	63.91	0.16769	0.1701	15.22	554	*ING3*
8p23.1	8.56–8.86	0.3	0.23346	0.23346	54.35	1	
9p24.2	3.90–4.15	0.25	0.00044905	0.001869	23.91	1	
9p21.3	21.96–21.98	0.02	0.000024325	0.00047	32.61	2	*CDKN2A*
10p15.3	1.21–1.60	0.39	0.00085355	0.001942	26.09	1	
10p15.1	5.04–5.09	0.05	0.0098168	0.096819	28.26	1	*AKR1C2*
11q22.3	83.17–135.01	51.84	0.00052488	0.00452	30.43	400	*CHEK1, H2AFX, MRE11A, ATM*
11q23.3	72.39–135.01	62.62	0.00042996	0.09682	36.96	489	*CHEK1, H2AFX, MRE11A, ATM*
13q11	0–23.90	23.90	0.02593	0.028436	26.09	29	*GJB2, LATS2, CRYL1*
13q22.3	78.34–79.18	0.84	0.065075	0.064205	15.22	1	*EDNRB*
16q23.1	78.13–79.63	1.5	0.13003	0.12649	13.04	1	*WWOX*
18q22.3	51.06–78.08	27.02	0.00041581	0.00042	43.48	106	*BCL2*
19q12	30.20–30.41	0.21	0.0022153	0.001942	10.87	1	*CCNE1*
21q21.1	19.26–19.64	0.38	0.080113	0.08011	36.96	1	*CHODL*
21q22.3	44.30–44.40	0.1	0.043869	0.04088	39.13	1	*NDUFV3*

[a]Mb: mega base pair.

Table 3. Distinct copy number gains and losses identified from 68 OSCCs randomly extracted from GSE25103 data repository.

Gains				Losses			
Cytogenetic Loci	GISTIC Wide Peak region (Mb[a])	Size (Mb)	GISTIC q-Value	Cytogenetic Loci	GISTIC Wide Peak region (Mb)	Size (Mb)	GISTIC q-Value
2q11.2	97.5–97.62	0.12	0.00079806	1p31.3	64.24–64.61	0.37	0.0011493
7p11.2[b]	55.76–55.79	0.03	0.00041856	1p13.2	112.30–112.94	0.64	0.0089268
9p24.1	5.38–5.72	0.34	0.0068263	1q43	236.77–236.96	0.19	0.021557
11q13.3[b]	70.12–70.15	0.03	2.49E-09	1q44	245.86–247.05	1.19	0.087333
11q22.1	101.26–101.29	0.03	5.69E-07	2p22.3	0–243.20	243.20	0.086976
11q22.1	101.67–101.69	0.02	9.38E-06	2p22.3	0–243.20	243.20	0.064816
11q22.2	102.64–102.98	0.34	7.68E-05	2q21.2	133.42–134.89	1.47	2.82E-15
11q13.3	68.72–68.85	0.13	6.25E-05	2q22.1	139.66–143.64	3.98	0.045925
				2q32.1	186.70–187.46	0.76	0.0023355
				2q35	204.79–243.20	38.41	0.0023355
				2q33.3	204.83–222.29	17.46	0.004378
				3p26.3	0–72.90	72.90	0.0027105
				3p26.1	4.36–4.54	0.18	2.73E-05
				3p22.1	17.20–61.24	44.04	0.0023355
				4q25	109.08–109.54	0.46	0.079403
				4q35.2[b]	187.48–188.23	0.75	0.0023355
				5q11.2	58.26–59.79	1.53	0.0002868
				5q15	93.45–93.96	0.51	0.0069236
				5q33.1	150.87–151.04	0.17	0.13923
				5q34	159.91–160.36	0.45	0.051805
				6p21.1	41.18–41.22	0.04	0.060272
				7q31.1[b]	110.73–111.37	0.64	0.0076292
				7q36.1[b]	132.92–159.14	26.22	0.042691
				8p23.2	2.09–6.26	4.17	2.13E-11
				8p23.1	6.60–6.61	0.01	5.55E-05
				8q11.2	51.71–52.73	1.56	0.046378
				9p24.1	4.86–5.16	0.3	0.0001564
				9p24.1	8.31–12.69	4.38	0.0002017
				9p21.3	20.71–21.01	0.3	6.34E-05
				9p21.3[b]	21.96–21.98	0.02	1.84E-13
				10p15.3	1.21–1.60	0.39	2.20E-22
				10q23.32	0–135.53	135.53	0.20781
				11q24.3[b]	118.39–135.01	16.62	0.0023355
				16p13.2	9.33–10.48	1.15	0.045925
				16q23.2	80.58–81.01	0.43	1.20E-05
				16q23.3	82.20–83.84	1.64	0.043591
				17p12	10.74–11.50	0.76	0.019221
				18q12.2	21.90–78.08	56.18	0.010034
				18q21.32[b]	42.79–78.08	35.29	5.55E-05
				19p13.3	6.11–6.22	0.11	0.052629
				19q13.12	37.73–37.83	0.1	0.0066528
				21q22.3[b]	0–48.13	48.13	0.21923

	22q13.2	27.07–51.30	24.23	0.17385

[a]Mb: mega base pair. [b]CNA loci that also identified in the 12 common CNAs.

cancers

Table 4. Association between *EGFR* and *CCND1* genes CN status.

		CCND1 CN Status	
		Neutral	Amplification
EGFR CN status	Disomy	99 (39%)	79 (31%)
	Polysomy/amplification	23 (9%)	56 (22%)
